# Determining the Diagnostic Value of Venous Sinus Density Indices in Non‐Contrast Brain CT Scan for Early Diagnosis of Cerebral Venous Sinus Thrombosis

**DOI:** 10.1002/brb3.70324

**Published:** 2025-02-11

**Authors:** Mehdi Maghbooli, Mohammad Kermani, Seyed Nariman Tavakoli Sany, Melina Arfaei

**Affiliations:** ^1^ Department of Neurology, Faculty of Medicine Zanjan University of Medical Sciences Zanjan Iran; ^2^ Student Research Committee, Department of Neurology, Faculty of Medicine Zanjan University of Medical Sciences Zanjan Iran

**Keywords:** cerebral venous sinuses thrombosis, Hounsfield unit value, non‐contrast CT scan, non‐contrast computed tomography

## Abstract

**Background and Aim:**

A non‐contrast brain CT Non‐contrast computed tomography (NCCT) scan is a valuable and cost‐effective way to detect cerebral venous sinus thrombosis (CVST) during its acute phase. The goal of this study was to evaluate how effective this diagnostic approach is, including its various density indices, to enable a more precise and timely diagnosis of this debilitating condition.

**Method:**

This retrospective case‐control study was conducted on 88 patients with suspected acute CVST. We analyzed NCCT scans of patients with suspected CVST using a Bayesian regression model with a 95% confidence level. We also conducted ROC analysis in R4.1.0 to determine the optimal cut‐off point.

**Result:**

We discovered a significant variance in the average sinus attenuation when comparing patients with acute CVST (*p* < 0.001). By utilizing an optimal cutoff of 61 HU (Hounsfield unit), we achieved sensitivities of 77.1% and specificities of 92.5% for average sinus attenuation. In addition, the optimal cutoff for standardized parameters included the ratios of HU/H (Hematocrit), HU/ICA (internal carotid artery), HU/BA (basilar artery), HU/FRONTAL lobe, HU/TEMPORAL lobe, and HU–BA, which were 1.41, 1.52, 1.63, 1.6, 1.6, and 23, respectively (*p* < 0.001). Area under the ROC curve for absolute venous Hounsfield was 0.88.

**Conclusion:**

NCCT is a reliable diagnostic approach for acute cerebral venous sinus thrombosis in emergency scenarios. Standardized parameters of absolute Hounsfield unit venous sinus thrombosis increase diagnosis accuracy. It is suggested to use these parameters as a complement to each other for more accurate diagnosis.

## Graphical abstract

The study evaluated how effective non‐contrast brain CT (NCCT) is in diagnosing acute cerebral venous sinus thrombosis (CVST) in 88 patients. We found that using an optimal Hounsfield unit cut‐off of 61HU resulted in sensitivities of 77.1% and specificities of 92.5%. By applying standardized parameters, we were able to improve the accuracy of diagnosis, highlighting the reliability of NCCT in emergency situations.

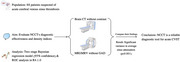

## 1 Introduction

Cerebral venous sinus thrombosis (CVST) is responsible for 0.5% of all strokes and occurs in three to four cases per million in the adult population (Guenther and Arauz 2011). CVST can have a variety of nonspecific symptoms, such as focal neurologic defects, seizures, headaches, and so on. Consequently, CVST is typically not diagnosed in the early stages (Stam 2005; Linn et al. 2009). With an approximate mortality rate of 8%–19%, CVST is known to be a contributing factor to stroke in children and infants (Wagner et al. 2015). Numerous imaging modalities are available for the diagnosis of CVST or any neurological disorders.

Non‐contrast computed tomography (NCCT) is the most popular imaging test for patients with nonspecific neurological manifestations who are taken to the emergency unit (Saposnik et al. 2011). The only apparent characteristic of CVST is a rise in venous sinus density (also known as the cord sign) (Avsenik et al. 2016). Therefore, detecting CVST in the early stages is extremely challenging and requires using additional methods of evaluation (Buyck et al. 2013).

Previous studies have examined the diagnostic accuracy of some NCCT imaging markers in the early detection of CVST, such as the Hounsfield unit (HU) index and its ratio to the hematocrit level of cerebral venous sinuses, and the ratio of the thrombosed venous sinus HU to the HU of the basilar artery (Avsenik et al. 2016; Canakci et al. 2021; Tanveer et al. 2023; Buyck et al. 2019; Tayyebi et al. 2020). However other density indices remain to be evaluated.

This study was conducted to evaluate the efficacy of brain NCCT and its multiple density indices, including—but not limited to—the ratio of HU to the average HU of the left and right internal carotid arteries, and the ratio of HU to white matter parenchyma in the frontal and temporal lobe, to help diagnose acute CVST more accurately and quickly.

## 2 Method

### 2.1 Study Design and Patient Selection

This retrospective case‐control study was conducted on patients suspected of acute CVST, who were admitted to the neurology clinic of Valiasr Hospital in Zanjan—as the only neurology center in the province—between March 2019 and September 2022.

The definitive diagnosis of acute CVST was determined based on magnetic resonance venography (MRV). Information regarding venous sinus density on non‐contrast brain CT scans was collected according to HU and standardized density indices, and then analyzed statistically.

The NCCT scans were performed using a multi‐slice Siemens Healthineers CT scanner. The main CT parameters included: tube voltage of 120 kVp, tube current of 200–300 mA, slice thickness of 5 mm, reconstruction algorithm Standard, and a field of view (FOV) of 250 mm. Optimization of these parameters was undertaken to guarantee superior image quality and patient safety.

The images were analyzed using Syngo via VB20A software (Siemens Healthineers), which allowed for precise measurement of HUs and other relevant indices. The selection of the program was based on its accuracy in processing radiological images and its capability to meet the unique needs of this work.

**FIGURE 1:**
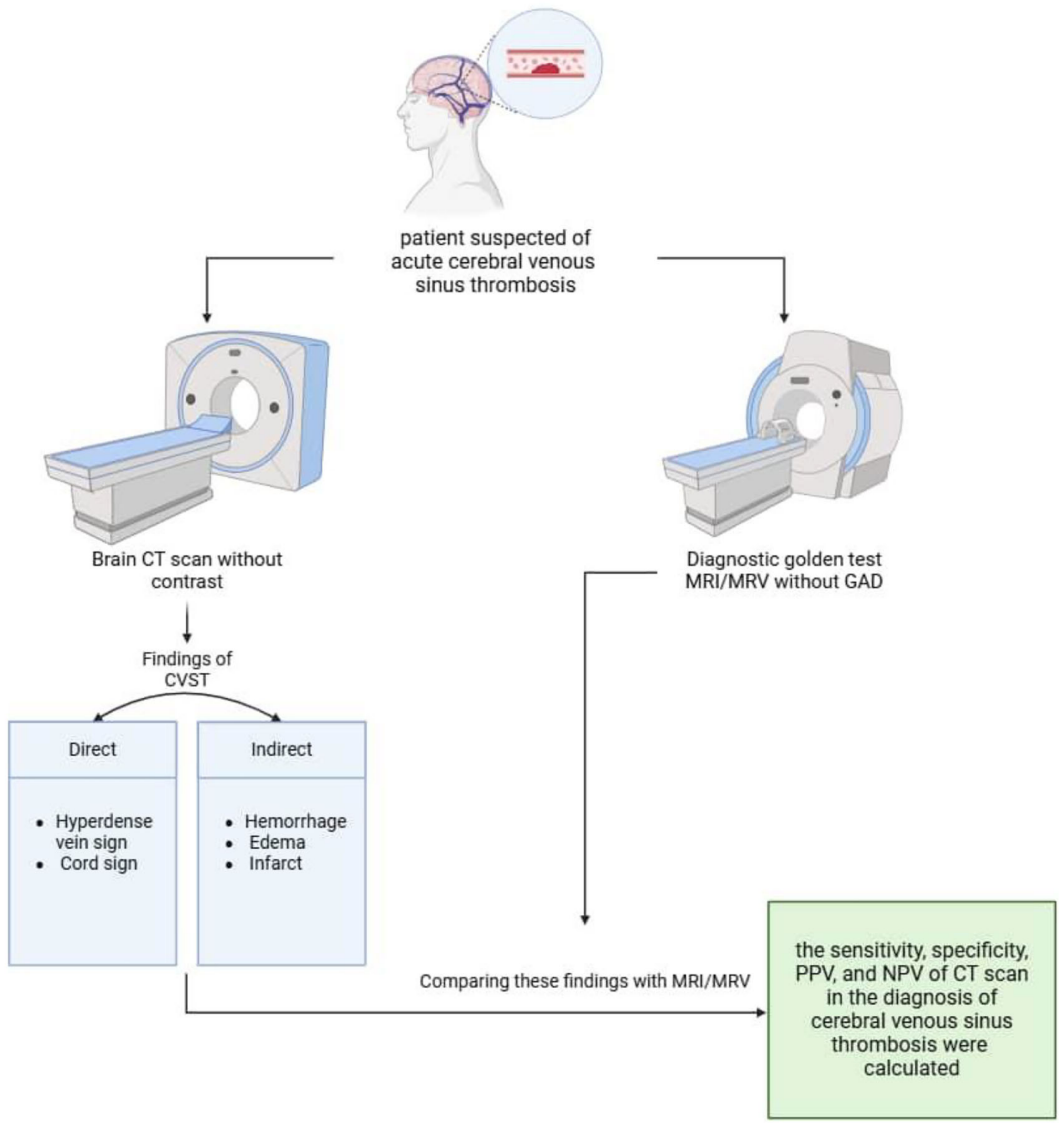
The process of diagnosing patients suspected of CVST and comparing the findings of diagnostic methods.

After receiving the ethics code (code: IR.ZUMS.REC.1400.520), information was extracted from the hospital's medical record system. At first, the number of samples and controls was determined based on statistical formulas, and then the samples were collected and analyzed based on the inclusion criteria. The case and control groups were matched based on age, gender, hemoglobin, hematocrit, blood urea nitrogen (BUN), and creatinine (Cr). The CT scans conducted for the control group were not specifically carried out for the objectives of this study. Retrospectively, the control data were obtained from patients who had previously received CT scans for reasons unrelated to CVST, such as headaches, mild injuries, or neurological complaints. These individuals received normal CT scans and were subsequently verified to be free of CVST, either clinical follow‐up or advanced imaging (Figure 1).

Samples were collected from patients who were hospitalized with a possible diagnosis of CVST and who had early (in the first 24 h of admission) brain CT scan without injection, as well as MRV/MRI without gadolinium.

Then, based on the direct (cords signs, dense vein sign) and indirect (edema, bleeding) findings of CVST in NCCT and comparing it with MRV/MRI (without gadolinium), the sensitivity, specificity, positive predictive value (PPV), and negative predictive value (NPV) of CT scan in the diagnosis of CVST were calculated.

HU measurements were conducted by drawing regions of interest (ROIs) on the venous sinuses in the NCCT images. The ROIs were positioned to include the largest possible cross‐sectional area of the sinus, while excluding nearby structures. This approach aimed to reduce any potential bias and inconsistency in the data.

In order to reduce any bias in the data‐collecting procedure, a blinded observer who was unaware of the patient groups (case or control) did the HU measurements. In order to ensure the precision and dependability of the measurements, the observer had 2 years of expertise in radiological evaluations.

In the next step, patients with a confirmed diagnosis of CVST underwent a brain NCCT to evaluate venous sinus density (according to HU value) and its standardized values. Standardized values are given in Table 1.

**TABLE 1:** Standardized parameters of the Hounsfield unit value.

Standardized parameters	Abbreviations
Ratio of the absolute Hounsfield unit value of the venous sinus to the hematocrit	HU/HCT
Ratio of the Hounsfield unit of the venous sinus thrombosis to the average Hounsfield unit of the left and right internal carotid arteries after their exit from the carotid siphon	HU/ICA
Difference between absolute Hounsfield unit of the venous sinus from the Hounsfield unit of pontine basilar artery	HU–BA
Ratio of the thrombosed venous sinus Hounsfield unit to the Hounsfield unit of the basilar artery in front of the pons	HU/BA
Ratio of venous absolute Hounsfield unit to white matter parenchyma in the frontal lobe	HU/FL
Ratio of venous absolute Hounsfield unit to white matter parenchyma in the temporal lobe	HU/TL

Inclusion criteria included:

1. Adult patients ≥ 18 years old with no prior history of CVST.
2. Taking a brain NCCT within 24 h of admission following clinical examination and history, as per CVST guidelines.
3. CVST confirmation with MRV/MRI (without gadolinium) as the reference standard within 10 days of the NCCT.
4. Taking hematocrit, BUN, and Cr within 24 h of admission.

Exclusion criteria included:

1. Patients presenting with focal neurological symptoms, seizures, symptoms of increased intracranial pressure, or altered consciousness, whose brain CT scan is scheduled more than 2 weeks after symptom onset.
2. Images with low quality and containing artifacts.
3. Patients with a history of trauma and brain surgery procedures within the last 2 weeks of brain CT scan.
4. The presence of old venous sinus thrombosis, the presence of blood in the extra‐axial space, intra‐axial, and extra‐axial brain masses in the vicinity of the venous sinus, and hypoplastic sinuses.
5. Patients with a skull fracture in the vicinity of venous sinuses.
6. Patients who received blood at the time of hematocrit evaluation and brain CT scan.

### 2.2 Statistical Analysis

After evaluating the venous density and comparing it with the control group using statistical formulas, we calculated statistically significant diagnostic cutoffs for diagnosing CVST. We also calculated the sensitivity, specificity, PPV, and NVP for each of the standardized criteria used to diagnose CVST.

The data were entered into SPSS version 28 software. Descriptive statistics and graphs were used to analyze the variables based on their type after the initial review and determining the distribution of the investigated variables.

To compare the means of the parameters between the patient and control groups, the independent samples *t*‐test was applied to normally distributed data, as confirmed by the Kolmogorov–Smirnov test. For data that were not normally distributed, the Mann–Whitney *U* test was used. Univariate methods were used at a 90% confidence level to examine how the investigated factors relate to the two‐stage variable of VST. We utilized a Bayesian two‐stage regression model at a 95% confidence level to explore the connection between the factors under investigation and the occurrence of venous sinus thrombosis. This analysis accounted for the influence of other independent, background, and confounding variables. Also, receiver operating characteristic (ROC) curve analysis was used to determine the optimal cut‐off point for predicting venous sinus thrombosis. Area under the curve (AUC) values, sensitivity, specificity, PPV, and NPV were investigated. All the above analyses were performed in the R4.1.0 environment and using the relevant software packages.

## 3 Results

### 3.1 Descriptive Statistics

In this study, we enrolled 88 patients who were suspected to have acute CVST. Out of these 88 patients, 48 cases (54.5%) were diagnosed (by the gold standard tests) with acute CVST, including 30 women (62.5%) and 18 men (37.5%). In the control group, out of 40 people, 27 cases (67.5%) were female and 13 cases (32.5%) were male. The ages of patients diagnosed with CVST ranged from 20 to 75 years. Among them, 31 patients (35.4%) were between the ages of 30 and 39 years.

After matching for age and gender, taking into account Cr and BUN levels as indicators of dehydration, a total of 40 individuals were selected for the control group. Analysis showed that there was no statistically significant difference between the case and the control groups in terms of gender (*p* = 0.934) and mean age (*p* = 0.971). Since dehydration can affect the amount of venous sinus density and lead to a false‐positive result, Cr and BUN were used as a measure of dehydration, which statistically showed that there was no significant difference in terms of Cr (*p* = 0.293) and BUN (*p* = 0.427) between the case and the control group.

The NCCT scan was divided into two general categories: normal and suspected venous sinus thrombosis. Suspicious cases were classified according to direct signs (hyperdense vein sign and cord sign), indirect signs (hemorrhage, edema, and infarct), and the simultaneous presence of both direct and indirect signs.

Out of the 88 participants, 61 cases (69.3%) showed a normal brain CT scan, while 27 cases (30.7%) showed signs of venous sinus thrombosis. Among these 27 cases, 19 had direct signs (21.6%), 3 had indirect signs (3.4%), and 5 displayed both direct and indirect signs at the same time (5.7%).

Also, as a gold‐standard diagnostic test, MRV/MRI was performed in all participants, which was a definite indicator of thrombosis in 48 patients (54.5%) and was normal in the other 40 patients (45.4%).

In the next step, standardized parameters of an absolute venous HU were investigated in the patients diagnosed with acute venous sinus thrombosis (matched with the control group).

The agreement between NCCT and gold‐standard tests (MRV/MRI) was 41%. To measure the agreement between these two tests, the kappa statistic was used, and its value was 0.1132, which indicates a weak agreement between these two tests. The kappa statistic was calculated by comparing the diagnostic outcomes of NCCT and MRI/MRV for the same patients, taking into account both positive and negative results. A brain CT scan without contrast to diagnose VST in the acute stage had 41.7% sensitivity, and 82.5% specificity, and its positive and negative predictive values were 74.1% and 54.1%, respectively.

In the next step, for early diagnosis of VST in the acute stage, a quantitative evaluation of the absolute HU and providing an optimal cutoff for venous sinus thrombosis was done. Then, the distribution of the anatomical location of the venous sinus with thrombosis was evaluated, and the left transverse sinus was the most frequent anatomical location for the thrombosed venous sinus (25.5%).

### 3.2 Density Measurement Parameters and Optimal Cutoffs

The statistical analysis determined that the optimal cutoff was ≥ 61 HU, with corresponding PPV = 92.5%, and NPV = 77%, resulting in a sensitivity of 77% and a specificity of 92.5%. Therefore, there was a significant statistical relationship between this parameter and VST (*p* < 0.001). In the patient group, the absolute HU ranged from 51 to 74, with an average of 63.917 HU. In the control group, the range was 48–66, with an average of 55.1 HU. These findings indicate a significant difference between patients and healthy controls (*p* < 0.001).

The HU/HCT ratio differences ranged from 1.1 to 3.15 (mean 1.682), which was significantly higher in the patient group. The HU/ICA ratio had an optimal cutoff of 1.52 and a range of 1.27–2.72 (mean 1.643). The HU/BA ratios ranged from 1.23 to 3.01 (mean 1.698), and the HU–BA difference between the venous sinus with thrombosis and the HU of the basilar artery in front of the cerebral pons had a range of 10–46 with an average of 25.917 in patients. HU/F resulted in a range of 1.340–2.1. Finally, the HU/T ratio ranged from 1.330 to 2.1 and showed a significantly higher amount (*p* < 0.001) compared to healthy controls, as represented in other parameters.

**FIGURE 2:**
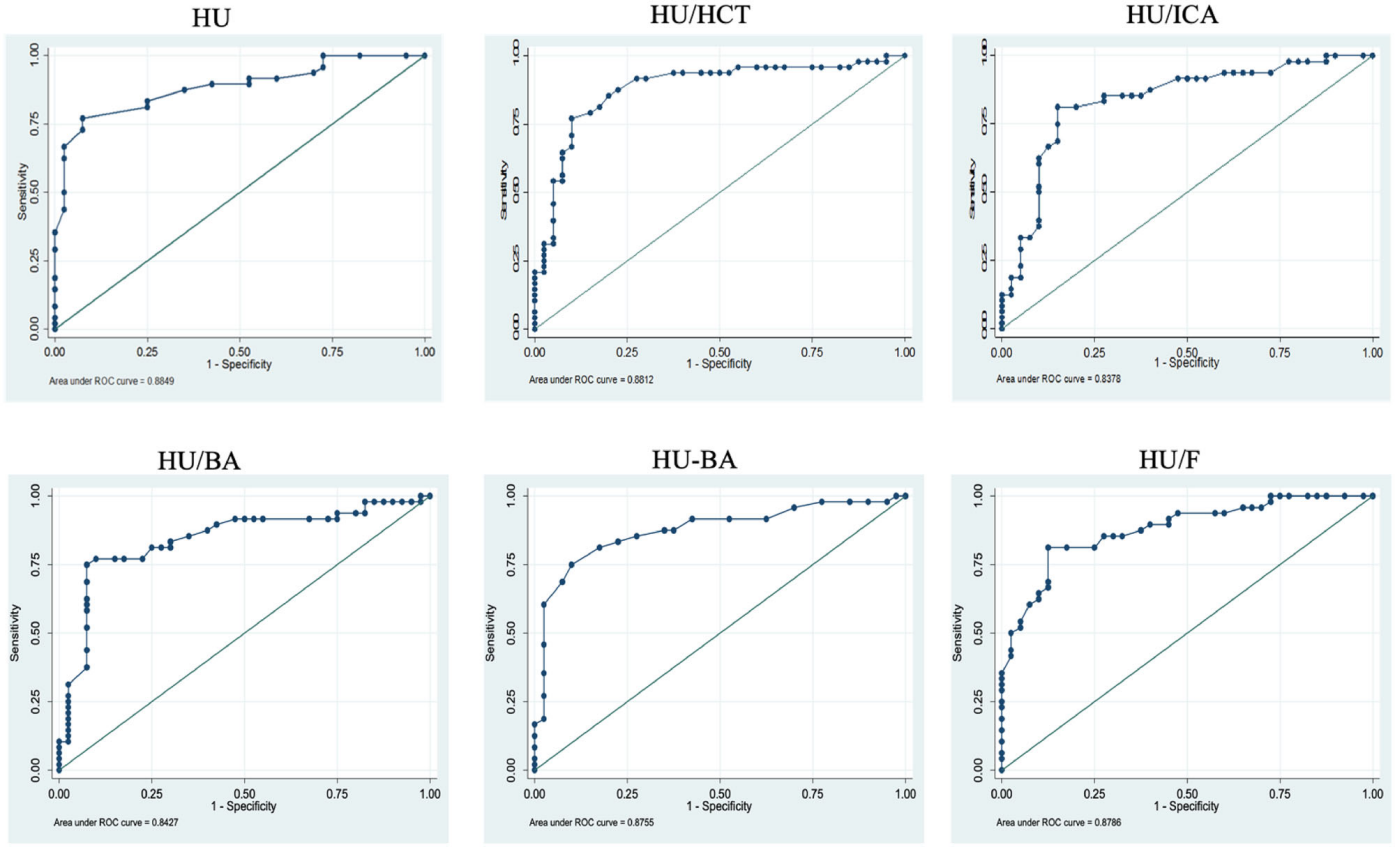
The area under the ROC curve for the standardized parameters.

Absolute HU values, HF/HCT ratio, HF/ICA ratio, HF/BA ratio, HF–BA difference, HF/F ratio, and HF/T ratio showed significant correlations for VST detection. These parameters displayed differences between patients and controls, indicating their potential for identifying VST. All the parameters had a strong association with VST (*p* < 0.001), establishing their importance in diagnosing this condition (Figure 2).

While demographic and basic clinical parameters show no significant differences, the imaging characteristics (HU values) reveal notable differences between the case and control groups, which could be important for diagnosis or understanding the underlying pathology (Tables 2 and 3).

**TABLE 2:** Diagnostic values of non‐contrast brain CT scan for acute stage of CVST.

Index	Sensitivity	Specificity	PPV	NPV	Optimal cutoff	*p* values	Range of Hounsfield unit value		Average of Hounsfield unit value	
							Patient	Control	Patient	Control
NCCT	41.7%	82.5%	74.1%	54.1%	≥ 61	< 001/0	51–74	48–66	63.917	55.1
HU	77.1%	92.5%	92.5%	77.1%	≥ 61	< 001/0	51–74	48–66	63.917	55.1
HU/HCT	91.7%	72.5%	80%	87.9%	≥ 1.41	< 001/0	1.1–3.15	1.05–1.83	1.682	1.338
HU/ICA	81.3%	85%	86.7%	79.1%	≥ 1.52	< 001/0	1.27–2.72	1.15–1.83	1.643	1.413
HU–BA	81.3%	82.5%	84.8%	78.6%	≥ 23	< 001/0	10–46	9–30	25.917	17.425
HU/BA	77.1%	90%	90.2%	76.2%	≥ 1.63	< 001/0	1.23–3.1	1.22–1.83	1.698	1.461
HU/FL	81.3%	87.5%	88.6%	79.5%	≥ 1.6	< 001/0	1.34–2.1	1.22–1.72	1.682	1.447
HU/TL	68.8%	95%	94.3%	71.7%	≥ 1.6	< 001/0	1.33–2.693	1.2–1.78	1.693	1.440

**TABLE 3:** Characteristics of patients with CVST and control group.

Variables		Case group, *N* = 48	Control group, *N* = 40	All patients, *N* = 88	*p* value
Gender	Male	18 (37.5%)	13 (32.5%)	31 (32.5%)	*p* > 0.05
	Female	30 (62.5%)	27 (67.5%)	57 (67.04%)	*p* > 0.05
Age		059.15 ± 417.40	745.15 ± 300.40	285.15 ± 364.40	*p* > 0.05
Hemoglobin		424.2 ± 040/13	687.1 ± 103/14	176.2 ± 523.13	*p* > 0.05
Hematocrit		388.5 ± 744.38	835.3 ± 360.41	898.4 ± 933.39	*p* > 0.05
BUN		15.798 ± 7.429	12.665 ± 5.842	14.374 ± 6.898	*p* > 0.05
Creatinine		0.923 ± 0.287	0.865 ± 0.214	0.897 ± 0.257	*p* > 0.05
Absolute Hounsfield unit value		63.917 ± 5.535	55.1 ± 4.673	59.909 ± 6.77	*p* < 0.05
HU/HCT		1.682 ± 0.32	1.338 ± 0.168	1.525 ± 0.312	*p* < 0.05
HU/ICA		1.643 ± 0.269	1.413 ± 0.137	1.539 ± 0.247	*p* < 0.05
HU/BA		1.698 ± 0.262	1.461 ± 0.163	1.59 ± 0.244	*p* < 0.05
HU–BA		25.917 ± 6.021	17.425 ± 4.771	22.057 ± 6.919	*p* < 0.05
HU/F		1.682 ± 0.149	1.447 ± 0.14	1.575 ± 0.186	*p* < 0.05
HU/T		1.693 ± 0.152	1.44 ± 0.141	1.578 ± 0.194	*p* < 0.05

## 4 Discussion

We found that the density of CVST in patients previously diagnosed with thrombosis by MRV—as the gold‐standard diagnostic—is different from healthy individuals. MRV and CTV are widely regarded as the gold standard imaging modality for detecting acute CVST due to its ability to identify blood clots causing obstructions in the cerebral sinuses or veins (Saposnik et al. 2011; Ferro et al. 2017). However, the accessibility of MRV in emergency situations is limited, and it is often susceptible to artifacts and flow‐related interruptions (Shayganfar et al. 2019).

In our study, 27 patients exhibited both direct and indirect signs of VST. However, when using the MRV method, 48 patients were conclusively diagnosed with VST, indicating that the lack of direct and indirect symptoms does not exclude the possibility of VST. Similarly, Linn et al. (2009) found that the absence of direct signs does not exclude VST. The increase in sinus density due to obstruction, especially in the first week of the disease, is influenced by the levels of hemoglobin and hematocrit in the blood, so that an increase in hematocrit leads to false‐positive results (Lee et al. 2013). Therefore, we relied on the HU ratio to hematocrit (HU/HCT) as a more reliable measure; our results showed that this ratio had a sensitivity of 72.5% with an optimal cutoff of 1.41, and there was a significant relationship between this parameter and VST (*p* < 0.001). Similar to our findings, different studies have suggested various optimal cutoff for the HU/HCT ratio, ranging from ≥ 1.4 to 1.52. All three studies reported a sensitivity of over 80% for this parameter (Buyck et al. 2013; de la Vega Muns et al. 2019; Alsafi et al. 2015). Also, in a study by Besachio et al. (2013), it was found that a HU/HCT ratio exceeding 1.7 can be a valuable indicator for diagnosing VST. This ratio facilitates quicker diagnosis and reduces the necessity for additional diagnostic assessments.

In terms of the frequency distribution of the anatomical regions affected by thrombosis, our study identified the left transverse sinus as the most frequent site (25.5%), followed by the superior sagittal sinus (17%), and the right transverse sinus (17%). In previous studies also, the superior sagittal sinus and the right transverse sinus were found to be the most commonly affected anatomical regions by thrombosis (Alsafi et al. 2015; Ferro et al. 2004; Teasdale 2000). Factors contributing to the involvement and thrombosis of the superior sagittal sinus are its extended length and narrower diameter. Furthermore, the left transverse sinus is often considered hypoplastic compared to the right transverse sinus, which increases the likelihood of reduced blood flow velocity and subsequent thrombus formation (Avsenik et al. 2016).

Our study's findings are consistent with the findings of previous studies regarding the efficacy of NCCT in the diagnosis of CVST. In a study conducted by Roland et al. (2010), it was found that NCCT had a 75% sensitivity in the diagnosis of acute CVST. However, three true‐positive cases were identified through indirect indications without any sinus hyperdensiy. Eliminating these cases would result in a sensitivity drop of 62%. In a study conducted by Rizzo et al. (2010), the sensitivity and specificity of NCCT were 53% and 78%, respectively. In a review study conducted by Avsenik et al. (2016), the sensitivity of NCCT scans for the diagnosis of acute CVST is from 25% to 65% in various studies.

In our research, upon analyzing quantitative and standardized parameters in relation to the absolute HUs, we found a sensitivity rate of 77% for the higher optimal cutoff of 61 HU. This statistically indicates a significant relationship between this parameter and VST. Several previous studies have demonstrated that a HU exceeding 50 exhibits high sensitivity, making it valuable for diagnosing venous thrombosis (Avsenik et al. 2016; Buyck et al. 2013; de la Vega Muns et al. 2019; Zaheer et al. 2016; Fanous et al. 2010; Mubarak and Nizamani 2017).

Since the use of the standardized parameter of absolute venous HU ratio to hematocrit requires obtaining a blood sample from the patient, various studies have tried to implement this standardization with the help of other parameters such as basilar artery density, and so on. In our study, these standardization methods were examined and we tried to provide an optimal cutoff for each of these parameters. One of these parameters is the ratio of the HU of the VST to the average HU of the left and right ICA (HU/ICA), which was investigated in our study and the results were mentioned. In the study of Zaheer et al. (2016), for this parameter (HU/ICA), the optimal cutoff of ≥ 1.5 with a sensitivity of 81% was presented.

The next parameter is the HU/BA ratio. In a study conducted by Tayyebi et al. (2020) in Iran on 50 patients with CVST, it was found that the optimal cutoff of ≥ 1.43 for this parameter (HU/BA) in the diagnosis of thrombosis had 100% sensitivity and 78% specificity (*p* < 0.001).

According to our findings, considering the cases of false negatives and false positives, as well as the sensitivity of NCCT in the diagnosis of acute CVST, this modality is insufficient for diagnosis and there is a need for more accurate diagnostic tests such as MRV or CT venography. Previous studies also supported this conclusion (van Dam et al. 2020; Hedderich et al. 2019).

In cases of VST with a normal NCCT, the presence of lateral venous channels, recanalization of blocked veins, and slow clot formation are among the contributing factors (Rizzo et al. 2010).

### 4.1 Strength and Limitation

The methods used in our study were NCCT scan and MRV/MRI without the use of gadolinium. Due to the harm of gadolinium in patients with kidney problems, these diagnostic methods can be used in such patients for the diagnosis of CVST. Also, our research indicates that the HU values exhibit a higher degree of reliability in comparison to other measured variables.

It is important to note that our study had limitations, which should be considered when interpreting the result. As a single‐center study, the sample size was relatively small (88 patients) and only included patients from a specific geographic region and ethnicity. Therefore, caution should be taken when generalizing the findings to other populations.

## 5 Conclusion

In emergency scenarios, the diagnosis of acute CVST is often challenging, particularly in cases where access to more accurate diagnostic methods such as MRV/MRI is limited. In such situations, brain CT scan without contrast—a cheap and available test—can be utilized as a reliable diagnostic approach. We discovered that by measuring the absolute sinus attenuation and the ratio target sinus/lowest attenuation sinus, we were able to enhance the sensitivity of NCCT in detecting superficial VST. We found that a sinus attenuation cutoff of > 62 HU or the ratio target sinus/lowest attenuation sinus > 1.3 were the optimal cutoffs for sensitivity. However, these findings still require validation in a prospective cohort. Our results support previous findings that visual assessment of a hyperdense sign has limited sensitivity, and this does not improve significantly with the use of multiplanar reformatted images. Also, standardized parameters of absolute HU VST can lead to quantification, increasing the accuracy of diagnosis and reducing the distorting effect of hematocrit. Since each of these standardized parameters has a unique level of sensitivity and specificity, it is recommended to use these parameters as a complement to each other for more accurate diagnosis. This method can provide physicians with a precise and timely diagnosis, allowing them to make informed decisions regarding the course of treatment.

## Author Contributions

**Mehdi Maghbooli**: conceptualization, methodology, validation, investigation, formal analysis, data curation, supervision, resources, visualization, writing–original draft. **Mohammad Kermani**: methodology, validation, investigation, conceptualization, formal analysis, visualization. **Seyed Nariman Tavakoli Sany**: writing–original draft, writing–review and editing, project administration, data curation, validation, methodology. **Melina Arfaei**: writing–review and editing, writing–original draft, investigation.

## Ethics Statement

This paper has been approved by the Ethical Committee of Zanjan University of Medical Sciences (Code: IR.ZUMS.REC.1400.520).

## Conflicts of Interest

The authors declare no conflicts of interest.

### Peer Review

The peer review history for this article is available at https://publons.com/publon/10.1002/brb3.70324

## Data Availability

All the data reviewed in this article are accessible.
